# Dominance of cropland reduces the pollen deposition from bumble bees

**DOI:** 10.1038/s41598-018-31826-3

**Published:** 2018-09-17

**Authors:** Sonja C. Pfister, Philipp W. Eckerter, Julius Krebs, James E. Cresswell, Jens Schirmel, Martin H. Entling

**Affiliations:** 1 University of Koblenz-Landau, Landau, Germany; 2 University of Exeter, Exeter, UK

## Abstract

Intensive agricultural landscapes can be hostile for bees due to a lack of floral and nesting resources, and due to management-related stress such as pesticide use and soil tillage. This threatens the pollination services that bees deliver to insect-pollinated crops. We studied the effects of farming intensity (organic vs. conventional, number of insecticide applications) and availability of semi-natural habitats at the field and landscape scale on pollinator visits and pollen delivery to pumpkin in Germany. We found that wild bumble bees were the key pollinators of pumpkin in terms of pollen delivery, despite fivefold higher visitation frequency of honey bees. Critically, we observed that the area of cropland had stronger effects on bees’ pollen deposition than the area of seminatural habitats. Specifically, a 10% increase of the proportion of cropland reduced pollen delivery by 7%. Pumpkin provides a striking example for a key role of wild pollinators in crop pollination even at high numerical dominance of honey bees. In addition, our findings suggest that habitat conversion to agricultural land is a driver of deteriorating pollination. This underlines the importance to maintain sufficient areas of non-crop habitats in agricultural landscapes.

## Introduction

Pollination is an important ecosystem service, especially for pollinator-dependent crops such as pumpkin^1^. Worldwide 75% of our leading food crops benefit from or even depend upon animal pollination^1^, translating into an annual value of pollination services around 235–577 billion US $^2,3^. Pollinator-dependent crops are mainly fruits, nuts and vegetables, which contain essential micronutrients. Therefore, pollination deficits can increase malnutrition^4^. Although their proportion of the global food volume is small (5–8%), the dependency of global food production on pollination is now twofold higher than fifty years ago^3^. At the same time, managed and wild bees declined globally owing to habitat loss, pesticides, mismanagement of bees, climate change, diseases and their interactions^3,5,6^. As a result, many pollinator-dependent crops suffer from pollination instability and deficit^7,8^. By enhancing the visits of bees and especially of wild bees, fruit set and yield of these crops can be increased^7,8^. Pollination intensity is usually measured as fruit or seed set or yield^7–10^. However, measures of natural pollen deposition should be better suited to distinguish among the potential drivers of pollination decline. First, it is more directly related to pollinator activity than fruit set and yield, which are influenced by many additional variables to pollination. Second, although pollinator activity is important, the amount of flower visits is not necessarily a good proxy for pollination, because flower visitors can vary largely in their effectiveness^7^. Nevertheless, there are few studies that relate actually measured, cumulative natural pollen deposition^11,12^ or yield^13^ to the suitability of agricultural landscapes for pollinator.

Wild bees are important pollinators, even in the presence of honey bees *Apis mellifera* Linnaeus 1758, because they ensure and enhance pollination through spatial and temporal complementarity, behavioural interactions and higher efficiency^7,10,14,15^. Further, the pollination services of wild bees can be consistent across fields with a similar landscape context, over days and years^16^. Pollinators need suitable habitats to persist in agro-ecosystems^17–19^. Therefore, environmental friendly farming practices and landscape management are needed to safeguard pollinators and pollination^3^. In general, wild bees need nesting sites and a continuity of abundant and diverse floral resources^6^. There is limited evidence for the importance of nesting resources^20^, whereas both flower richness and floral cover enhance the number of bee visits and diversity^21^. In addition, flower richness can contribute to a continuity of floral resources and thereby reduce temporal variability of bee visits^21^. Therefore, the abundance and diversity of wild bees are positively influenced by organic farming^22,23^ and seminatural habitats at the local and landscape scale^24,25^, which usually contain abundant and diverse flower resources i.a. owing to the absence of herbicides^26,27^. Seminatural habitats further provide nesting sites^20^. In consequence pollination is often more successful in organic than in conventional fields^28,29^ and in fields close to seminatural habitats^8–10^.

However, less is known about the negative effects of pesticides at the landscape scale. Wild bees are exposed to multiple pesticides^30,31^ and especially neonicotinoids can have adverse effects on bees^32,33^. Pesticides are frequently found in wild bumble bees, whereby more pesticides were found in bumble bees foraging in agricultural landscapes than in urban landscapes^31^. In addition to pesticide applications, habitat conversion to cropland has other negative effects on pollinators^6^. Frequent soil disturbance and vegetation removal prohibit nesting of wild bees in annual crops^6,20^. In addition, wind-pollinated crops such as cereals offer no floral resources to bees, especially if herbicides exclude wild flowering plants^6^. Even mass-flowering crops such as oilseed rape only offer monotonous resources for short time periods so that few, if any, bee species will be able to complete their life cycle on them^6,34^. Thus, although cropland includes some mass-flowering crops that offer some resources for bees for a limited time, cropland is a largely hostile environment for wild bee species, and land use change into crops may threaten their persistence in farmed landscapes^34^. Most existing studies do not distinguish among possible drivers of crop pollination at the landscape scale, namely the availability of seminatural habitats for nesting and alternative floral resources on the one hand, and potential negative drivers such as high proportions of cropland or the intensity of insecticide use in the surrounding landscape on the other^8,10^.

We addressed this gap of knowledge by studying pollinator activity and pollen delivery to pumpkin across replicated landscapes. We studied the combined effect of organic farming, field-bordering seminatural habitats (woody, herbaceous, or another field as control), land-use composition (proportion of cropland in 1 km radius) and insecticide intensity in the surrounding landscape (summed insecticide applications per crop weighted by crop area) on pollinator visits and pollen delivery in 18 pumpkin fields. We chose pumpkin, because pumpkin has separate male and female flowers and heavy pollen grains, thus it is obligate cross-pollinated by insects^35^. In addition, pumpkin has a short flower lifetime (6 hours – 1 day) and needs a high pollinating intensity (ca. 2,500 pollen grains are needed to maximize fruit set) and therefore effective and rapid pollinator visits^36–38^. We tested the following hypotheses:

1. Pollen delivery to pumpkin is related to the number of visits by honey and bumble bees.
2. The number of pollinator visits and thereby pollen delivery is higher in organic than in conventional fields and higher in fields with adjacent seminatural habitats compared to fields adjacent to another crop field (local management effects).
3. The proportion of cropland and insecticide intensity in the landscape reduce the number of pollinator visits and thereby pollen delivery (landscape effects).

## Results

In total we observed 2,100 bee individuals, of which 79% were honey bees *Apis mellifera*, 14% bumble bees (mainly *Bombus terrestris agg*., some *B*. *lapidarius*) and 7% halictid bees in 54 hours of video footage. At maximum 33,147 pollen grains were delivered to a female pumpkin flower, average delivery was 11,600 per flower (±5,692 s.d., n = 546).

**Figure 1:**
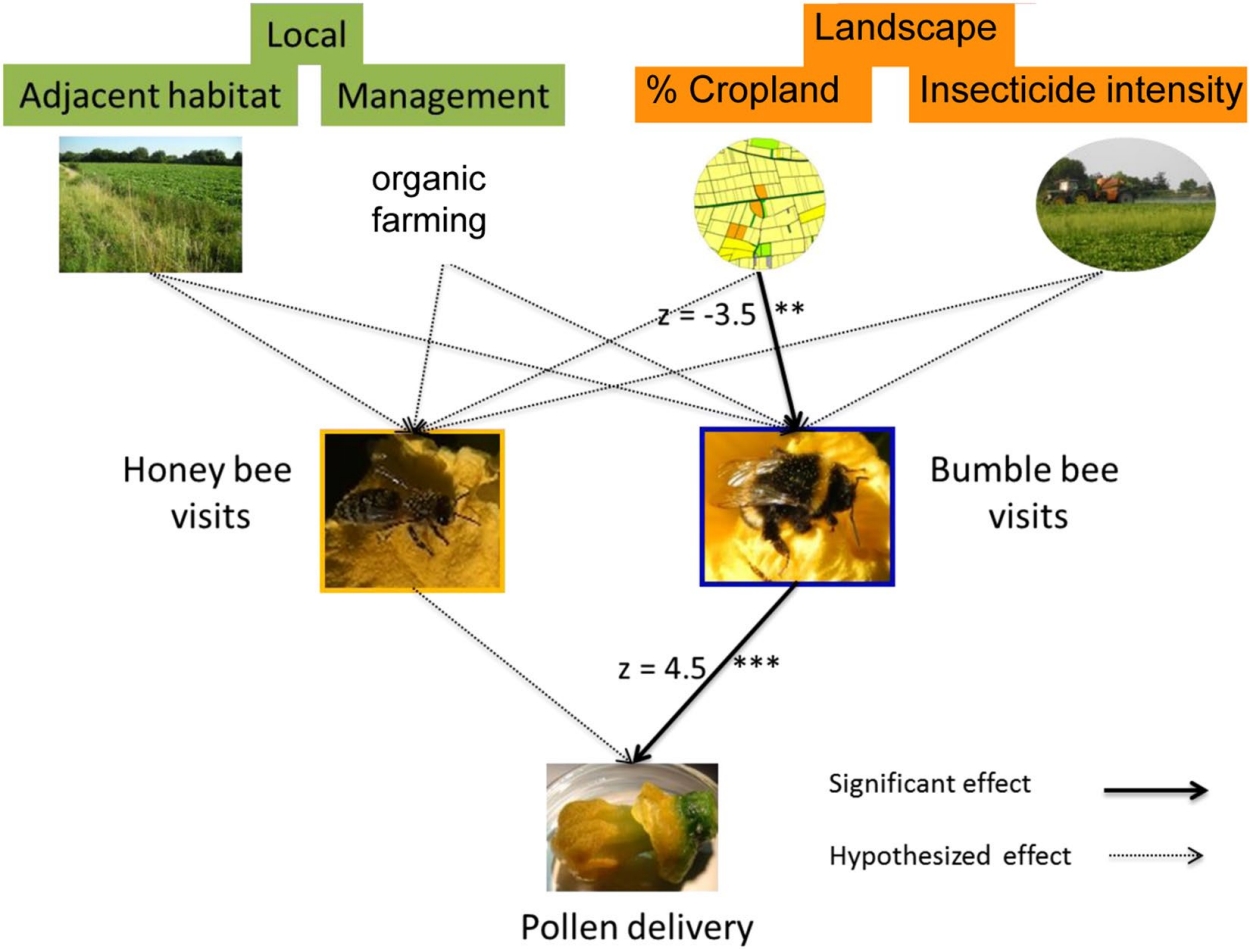
Effects on pumpkin pollination: Separation of the effects of adjacent habitat type (crop, SNH), management (organic, conventional), proportion of cropland in 1 km radius and insecticide intensity in the landscape on bumble bee and honey bee visits and the impact of all these variables on pollen delivery. Dotted arrows show hypothesised impacts, bold solid arrows show significant effects (p < 0.05) derived from the structure equation model. Proportion of cropland in 1 km radius decreased bumble bee visits. Pollen delivery only increased with bumble bee visits, but not with honey bee visits. Statistics see Table 1.

**Figure 2:**
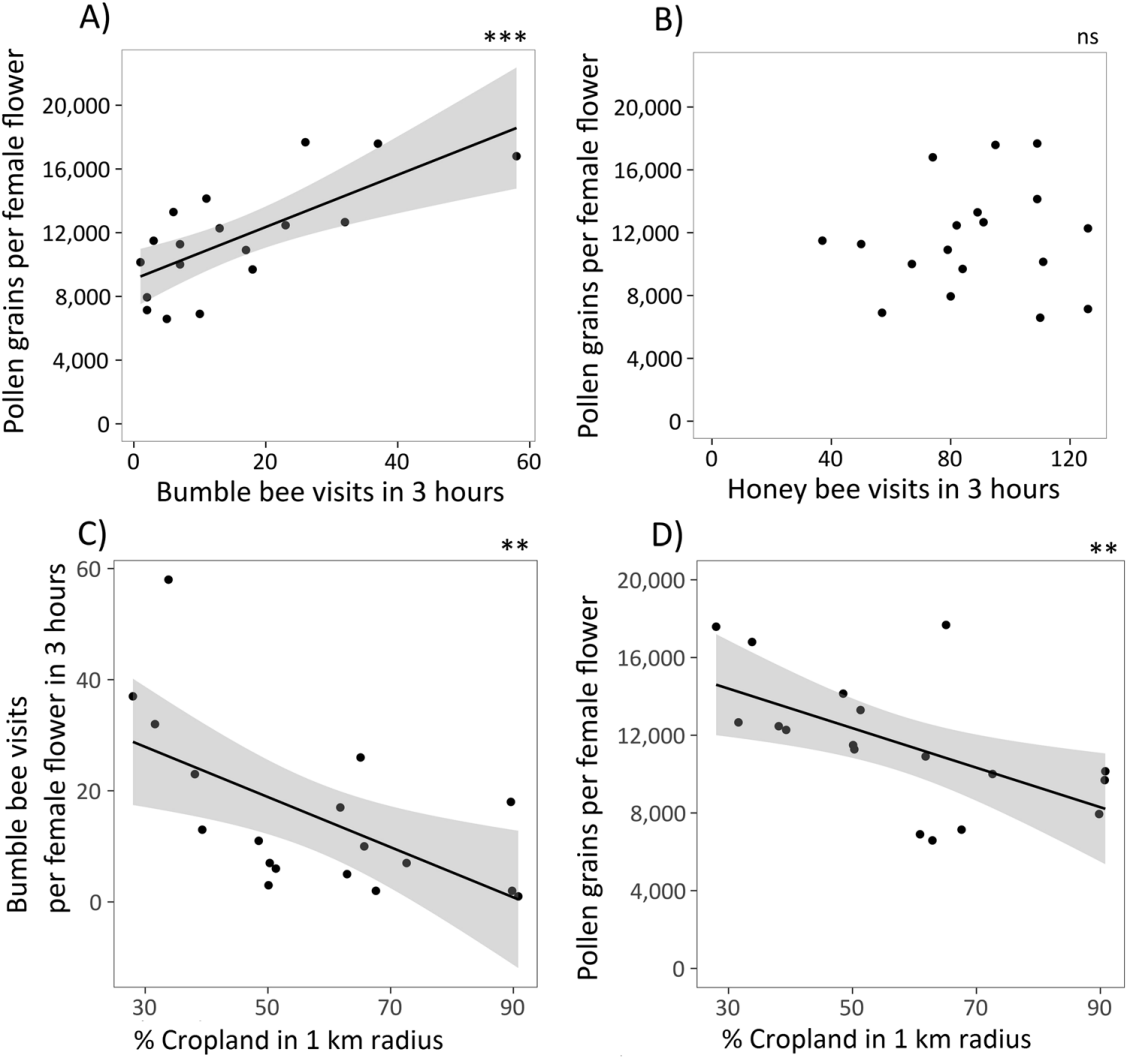
Pollen delivery increased with bumble bee visits (**A**), but was not related to the number of honey bee visits (**B**). The proportion of cropland in 1 km radius reduced the number of bumble bee visits (**C**) and pollen delivery (**D**). Statistics see Table 1.

Pollen delivery significantly increased with the number of bumble bee visits (p < 0.001), while the numerically dominant honey bees had no effect on pollen delivery (p = 0.38; Figs 1, 2A,B, Table 1). The proportion of cropland in 1 km radius strongly reduced the number of bumble bee visits (Fig. 2C, Table 1), with a corresponding decline in pollen delivery (Figs 1, 2D, Table 1). An increase of cropland in the surrounding landscape by 10% reduced the number of bumble bee visits by two and the number of delivered pollen grains by ca. 1,200 per female flower. The proportion of seminatural habitats tended to increase bumble bee visits and increased pollen delivery (second structural equation model see Table S4). However, the models with cropland were distinctly better than those with semi-natural habitats (pollen delivery: ΔAICc = 3.6, bumble bee visits: ΔAICc = 6.1). There were no significant effects of management, the adjacent habitat, and insecticide intensity on honey or bumble bees (Table 1).

**Table 1:** Direct effects of adjacent habitat (factor: crop or SNH), management (factor: organic or conventional), proportion of cropland in 1 km radius (% cropland, continuous), and insecticide intensity in the landscape (continuous) on visits of honey and bumble bees and direct and indirect effects of them on pollen delivery (hypothesised causal structure see Fig. 1).

Response	Mediated by	Predictor	Estimate	Std.Err	z-value	P	R^2^
Honey bee visits		~					0.33
		Adjacent SNH	12.1	21.2	0.6	0.57	
		Organic	−19.8	10.2	−1.9	0.053	
		% Cropland	0.1	0.4	0.3	0.76	
		Insecticide intensity	−4.2	3.8	−1.1	0.26	
Bumble bee visits		~					0.61
		Adjacent SNH	4.6	11.1	0.4	0.68	
		Organic	8.5	5.0	1.7	0.090	
		% Cropland	−0.64	0.19	−3.5	0.001	
		Insecticide intensity	0.3	1.8	0.2	0.88	
Pollen delivery		~					0.67
		Honey bee visits	22	25	0.9	0.38	
		Bumble bee visits	183	40	4.5	<0.001	
	Honey bee visits	Adjacent SNH	260	571	0.4	0.65	
		Organic	−425	523	−0.8	0.43	
		% Cropland	2	8	0.3	0.77	
		Insecticide intensity	−91	118	−0.8	0.45	
	Bumble bee visits	Adjacent SNH	829	2059	0.4	0.68	
		Organic	1566	973	1.6	0.11	
		% Cropland	−118	39	−3.0	0.003	
		Insecticide intensity	52	339	0.2	0.88	

Indirect effects on pollen delivery are split in effects mediated by bumble bee visits or by honey bee visits. Results from the structural equation model (number of observations = 18, minimum generalized least-squares chi-square statistic = 9.3, df = 11) are displayed. For all predictors estimates, standard errors, z-values and p-values are given. R^2^ is given per response.

## Discussion

Surprisingly, honey bee visits did not significantly contribute to pollen delivery in our pumpkin fields, although there were around five times more visits by honey bees than by bumble bees. This can partly be explained by the six times higher single-visit deposition of bumble bees compared to honey bees^38^. With on average 11,000 deposited pollen grains, around four times more pollen grains were deposited than needed to maximize fruit set (~2,500 pollen grains^38^). Thus, there is no pollination deficit in pumpkin in our region. Only 3% of the investigated flowers received less than 2,500 pollen grains, half of them because the flowers were filled with water from overhead irrigation. Nevertheless, crops with lower visitation rates per flower, such as strawberry, may suffer yield losses from pollination deficit^39^ (unpublished own data).

In contrast to our expectations, local management (organic farming and field-bordering seminatural habitats) had no significant effects on pollinator visits and consequently on pollen delivery. This may be owing to the large foraging ranges of honey and bumble bees^40^ in combination with the high attractiveness of pumpkin flowers. In late summer floral resources are scarce in agricultural landscapes^41^. Pumpkin flowers offer high nectar and sugar amounts (c. 290 µL nectar m^−2^ day^−1^ and 30 mg sugar m^−2^ day^−1^; amounts per flower^36^, combined with own flower density data). Consequently, pumpkin may attract honey and bumble bee populations from the wider landscape.

We hypothesized that organic farming has positive effects on pollinators and pollination mainly owing to two reasons. First, organic fields can favour pollinators through a higher weed cover and diversity of non-crop floral resources than conventional fields^26,27,42^. However, in contrast to these studies, conventional pumpkin fields in our study had a higher weed cover (8.5%) than organic fields (3.6%, Table S5). Although herbicides were applied in conventional fields (Table S4), conventional farmers can tolerate a higher weed cover in pumpkin, because pumpkin outcompete the weed and the weed pressure in subsequent crops can be regulated by herbicides, whereas organic farmers depend more strongly on low weed pressures for the subsequent crops and therefore mechanically removed the weeds.

Second, conventional farming could have adverse effects on pollinators through insecticides, especially neonicotinoids^32^. Still, the number of insecticide applications did not differ significantly between organic and conventional management in our study. However, management varied strongly within organic farming. Organic fields managed according to the EU-Eco regulation 834/2007 had more insecticide applications than conventional fields and organic fields managed by rules from organic associations, which ban insecticides completely (Table S5).

Future studies should, already in their design, consider more detailed farming practices to understand the responses of the beneficials^43^. Overall, the positive effects of organic farming on beneficial insects may have been overestimated owing to studies only including farms under very strict organic management without any pesticide use^26,28,44^. Thus, more studies comparing organic management according to the EU-Eco regulation 834/2007 with other managements are needed.

In line with Petersen & Nault (2014)^13^, the landscape effects on pollination were mediated by bumble bees. Surprisingly, the negative effects of cropland on bumble bee visits and pollen deposition were stronger than the positive effects of seminatural habitats. Several studies report positive effects of seminatural habitats, such as grassland^13^, forest^45^ or both^46^, on bumble bee visits and on modelled pollen deposition in pumpkin in North America and China. Similar to our findings, bumble bee visits and pollen deposition were higher in landscapes dominated by seminatural habitats (forest and grassland) and urban habitats than in landscapes dominated by agricultural land in North America^12^. As non-crop habitats (including seminatural habitats, urban habitats, water bodies and other habitats such as streets) were more important in our study than seminatural habitats alone, we conclude that although seminatural habitats are in general good habitats for pollinators, they often only make up a small part in agricultural landscapes and other habitats like urban green habitats or water bodies such as quarry ponds and their borders can also have positive effects on bumble bees or at least can buffer the negative effects of cropland. Existing studies of crop pollination in a landscape context did not distinguish between positive effects of seminatural habitats and negative effects of agriculture^8,10^. The dominant role of cropland cover in our study suggests that it should be included also in other studies of landscape management for ecosystem services. Surprisingly, the insecticide intensity in the surrounding landscape did not influence bee visits or pollen delivery. Thus, the hostility of agricultural landscapes seems to be mainly related to the lack of nesting sites and floral resources, which seem to be more important drivers than insecticide intensity. This can pose a dilemma to crop production if land conversion to crops creates a negative feedback on productivity via the decline of pollinators^47^. Hostility of cropland as the main limiting factor for pollination services implies that efforts of reducing farming intensity or adding only small surfaces of ecological compensation areas may offer little prospect of sustaining this ecosystem service in landscapes dominated by agriculture. Instead, a sufficient amount of non-crop habitats is needed. An appropriate management and a mixture of different non-crop habitat types help to improve their ability to provide nesting sites and continuous, abundant and diverse floral resources, and thereby their ability to promote pollinators^48^.

Our study demonstrates that honey bees, even at fivefold visitation frequency compared to bumble bees, have no measurable effect on pollen delivery to pumpkin. Thus, pumpkin provides a striking example for a dominant role of wild pollinators for pollination success of a crop. In addition, our study suggests that the dominance of cropland is the main limiting factor for the pollen delivery of pumpkin through its negative effect on bumble bee visitation. Thus, sufficient areas of non-crop habitats (such as seminatural or urban green habitats or water bodies) need to be maintained in agricultural landscapes for pollination-dependent crops.

## Methods

### Study sites

Pollinator visits and pollen delivery were studied in 2014 in 18 commercial pumpkin fields *Cucurbita maxima* Duchesne ex Poir cv Hokkaido (mean field size 3 ± 2.4 ha s.d.) in the Upper Rhine valley between Ludwigshafen and Kandel, Germany (49°4 N, 8°6 E; 49°27 N, 8°28 E; 90–155 m a.s.l.) (see Fig. S1 in Supporting Information). The region has a temperate climate with annual mean temperatures around 11 °C and 700 mm of annual precipitation on average (station Landau, German Weather Service). Each 9 fields were managed conventionally or organically (EU-Eco regulation 834/2007^49^), respectively. Pollen and pollinators were sampled along four transects per field at distances of 2, 10, 18, and 26 m from the edge to the field centre. In each six fields the field edge nearest to the sampling were another crop field, a herbaceous or a woody seminatural habitat (SNH). SNH were defined as any habitat containing a community of non-crop plant species and include herbaceous (e.g. field margins, fallows) and woody vegetation (e.g. hedgerows, forest fragments)^50^. In addition, the pumpkin fields were located in landscapes differing in the proportion of seminatural habitats (5–49%) and in the proportion of cropland in 1 km radius around the focal field (28–91%). Cropland includes annual and perennial crops, but excludes permanent pastures. Cropland in our study region mainly consisted of annual arable crops (winter wheat, maize, sugar beet, potato and different vegetables), but also included perennial crops like apples and rhubarb (see Tables S1, S2).

To calculate the proportions, we ground mapped habitats around the focal field in 1 km radius. The habitats were classified into 56 categories that included 45 crops (e.g. annual and perennial crop types, orchards), SNH (e.g. forests, grasslands, hedgerows and grass margins), urban areas, water bodies and other habitats. Any mapped element had a minimum width of 1.5 m and at least 50 m length and a minimum size of 75 m^2^. Land use classifications were confirmed with ground-truthing surveys at every site.

### Insecticide intensity

We used the landscape data of the 16 most abundant crop types to calculate an index of insecticide intensity for the landscape surrounding each field.

The landscape insecticide index *Ia* (*Eq*. *1*) was calculated by summing up the insecticide applications per crop *NI*_*k*_ weighted by crop area *% A*_*k*_ divided by the proportion of cropland in the landscape sector % *Aa* to have a value independent from the proportion of cropland in the landscape sector. *NI*_*k*_ is the average number of insecticide applications on crop k in conventional management according to the reports from the Federal Research Centre for Cultivated Plants^51–55^ and according to the regional extension service (Dienstleistungszentrum ländlicher Raum, pers. comm. 2016) (Table S2). For the number of insecticide applications on pumpkin we used the average value that was applied in 2014 and 2015 on 18 conventional pumpkin fields in the case study region (farmer’s questionnaires). *% A*_*k*_ is the proportion of the area occupied by crop k on the total area *A* of the landscape sector (314 ha).

### Pollinator visits

Flower visitors and their foraging behaviour were documented by a digital HD video camera recorder (handycam Sony ® HDR-CX115E). Each field was sampled at each one time period on three different days in July during the flowering period (2–6, 15–17, 23–25 of July 2014), once at 7:00, 8:30 and 10:00 am. All surveys were done in the morning, because pumpkin stigmas are only receptive in the morning^56^ and pollen grains are only viable^37^ and available^38^ then as well. On each occasion, we recorded four 15-minute-long videos, one point on each 2, 10, 18 and 26 m transect, each surveying a different female pumpkin flower. The camera was positioned ~50 cm above a female flower in order to monitor the mouth of the flower’s corolla. Weather conditions were comparable at all samplings (temperature at ground level 24 ± 5 °C [mean ± s.d.] measured by HOBO ® Pendant temperature/light data logger UA-002–08, wind velocity at 1.5 m above ground 0.8 ± 0.7 m/s [mean ± s.d.] measured by cup anemometer PCE-A420). From the videos we extracted the visitation rates for each bee group. Video recording is a suitable method to sample visitation rates in pumpkin^12,57^, because the frequency of visits is high and relatively evenly distributed across flowers. Three bee groups were distinguished: 1) honey bees *A*. *mellifera* L., 2) bumble bees = *Bombus terrestris* L. agg. (including *B*. *terrestris* Linnaeus 1758 and *B*. *lucorum* Linnaeus 1761) and *B*. *lapidarius* Linnaeus 1758 and 3) halictid bees (several species which could not be distinguished from the video data). Bee identification followed Schmid-Egger *et al*.^58^ and Amiet^59^.

### Pollen delivery

In order to quantify the pollen delivery by the collective pollinator fauna, we measured stigmatic pollen loads of open pollinated flowers corresponding to the first two samplings where we recorded the pollinator visits. In the 18 studied fields we harvested 16 stigmas per sampling and field (Σ 32/field) after 14:00 pm, when pollination had finished, and stored them in a freezer. We extracted the pollen by acetolysis following Jones^60^. After the acetolysis, glycerol 50% was added to the extracted pollen to a total volume of 2 mL. To evenly re-suspend the pollen, the vials were shaken by a vortex mixer prior to taking three subsamples of 50 µL from the pollen suspension. Each subsample was pipetted on 1 cm^2^ area and a picture (2560 × 1920 pixel) was taken by a microscope camera (Zeiss AxioCam ERc 5 s). The pollen on this picture was counted by image analysis (ImageJ v. 1.48, defined particle size 225–900 Pixel^2^, circulartiy 0.7–1.0). The total pollen load was extrapolated volumetrically from the mean of the subsamples.

### Statistics

We performed structural equation models (package “lavaan”^61,62^) in order to determine the effects of management (factor: organic or conventional), adjacent habitat (factor: SNH or crop), landscape insecticide intensity (continuous), proportion of cropland in 1 km radius (continuous), and proportion of SNH in 1 km radius (continuous) on flower visitation and pollen delivery. The proportion of cropland in 1 km radius and the proportion of SNH in 1 km radius were correlated (r = −0.65). Hence, a model with both variables would not be multivariate normal. Thus, we calculated two models, with either the proportion of cropland or the proportion of SNH plus the remaining explanatory variables. All numeric variables were tested for multivariate normality (package “MVN”^63^). With the structure equation models we studied the direct effects of the above mentioned explanatory variables on the number of honey or bumble bee visits in 3 hours [sum of 12 videos per field] and their indirect effects on pollen delivery on female pumpkin flowers [mean pollen delivery on 32 stigmas per field] mediated by honey and bumble bee visits. Covariances between the predictors were fixed, when they were independent from each other (pearson correlation r < 0.4, see Table S3). The following three covariances were not fixed: adjacent habitat and landscape insecticide intensity, adjacent habitat and proportion of cropland in 1 km radius, and proportion of cropland in 1 km radius and landscape insecticide intensity. Owing to our small sample size we used the generalized least-squares chi-square statistic^62^. Bonferroni corrections are overly conservative for structural equation models^64^. The probability of finding at least 3 nominally significant effects among 18 factors tested (see Table 1) by chance is only marginally higher than 5% (p = 0.058 according to Bernoulli equation^65^). Linear models relating bumble bee visits or pollen delivery with proportion of cropland were compared to models with proportion of seminatural habitats by Akaike´s information criterion for small sample sizes (AICc, package “AICcmodavg”^66^). All analyses were conducted in R 3.3.1^67^.

## Electronic supplementary material

Supplementary information

## Data Availability

The data supporting the results is archived in Figshare. DOI of the R Script for the Structural Equation Model: 10.6084/m9.figshare.7039991 DOI of the dataset analysed with the Structural Equation Model: 10.6084/m9.figshare.7040000.
