# Supplementary material for: Dominance of cropland reduces the pollen deposition from bumble bees
Source: Sci Rep. 2018 Sep 17;8:13873. doi: 10.1038/s41598-018-31826-3 (PMC6141495; doi:10.1038/s41598-018-31826-3)
Supplement: Supplementary file 1 — Supplementary information [file 41598_2018_31826_MOESM1_ESM.docx]

**Supplementary information**

**Dominance of cropland reduces the pollen deposition from bumble bees**

Sonja C. Pfister, Philipp W. Eckerter, Julius Krebs, James E. Cresswell, Jens Schirmel, Martin H. Entling

**Supplementary Figure S1, Supplementary Tables S1-S5**

**Supplementary Figure 1**


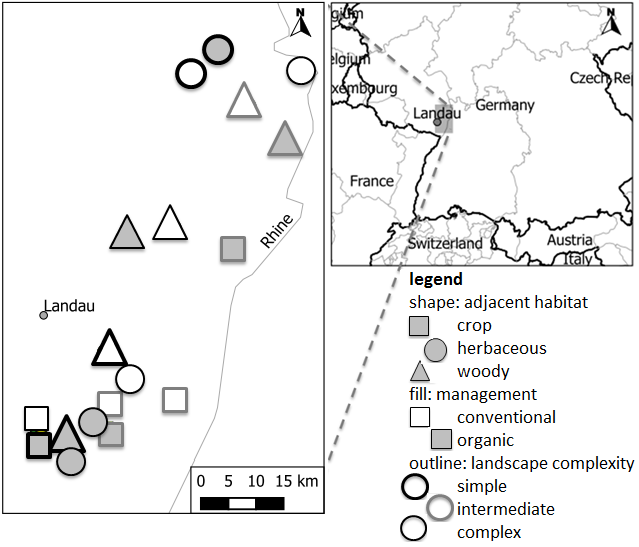


**Fig. S1** Geographic location of the 18 studied pumpkin fields in the Upper Rhine valley. Fields differed in adjacent habitats (crop, herbaceous, woody), management (organic, conventional) and landscape complexity in 1 km radius (simple < 10% SNH, intermediate 10-30% SNH, complex > 30% SNH in 1 km radius).

**Supplementary Tables**

**Table S1** Proportions of the different classified habitat types in 1 km radius around the 18 focal fields (landscapes = LS). In cropland the proportions of all annual and perennial crops are summed up. SNH is the sum of all seminatural habitats (ha = herbaceous areal, hl = herbaceous linear, wa = woody areal, wl = woody linear). In addition the farming system (organic: yes or no) and adjacent seminatural habitat of the focal field are given.

|  | **LS1** | **LS2** | **LS3** | **LS4** | **LS5** | **LS6** | **LS7** | **LS8** | **LS9** | **LS10** | **LS11** | **LS12** | **LS13** | **LS14** | **LS15** | **LS16** | **LS17** | **LS18** |
| --- | --- | --- | --- | --- | --- | --- | --- | --- | --- | --- | --- | --- | --- | --- | --- | --- | --- | --- |
| adjacent | crop | crop | herb | woody | herb | crop | crop | crop | herb | woody | woody | woody | crop | woody | herb | woody | herb | herb |
| organic | no | yes | yes | yes | yes | yes | no | no | no | no | yes | no | yes | yes | no | no | yes | no |
| **habitat** |  |  |  |  |  |  |  |  |  |  |  |  |  |  |  |  |  |  |
| Apple | 2.4 | 9.8 | 0.0 | 0.2 | 0.2 | 3.1 | 3.5 | 0.1 | 0.0 | 0.6 | 0.0 | 0.0 | 0.0 | 0.0 | 0.0 | 0.0 | 0.0 | 0.0 |
| Asparagus | 3.3 | 0.0 | 0.0 | 0.0 | 1.5 | 4.7 | 1.4 | 0.0 | 0.0 | 0.9 | 0.7 | 2.4 | 0.1 | 0.0 | 0.0 | 0.7 | 2.2 | 0.0 |
| Cabbage | 0.0 | 3.2 | 0.0 | 0.8 | 0.0 | 4.4 | 0.2 | 0.6 | 0.5 | 0.0 | 0.0 | 0.1 | 2.3 | 0.0 | 0.0 | 0.0 | 3.1 | 2.4 |
| Carrot | 0.0 | 0.0 | 0.0 | 0.0 | 0.0 | 0.0 | 4.0 | 4.7 | 2.2 | 0.7 | 0.0 | 0.5 | 0.2 | 7.1 | 0.0 | 1.3 | 3.0 | 4.6 |
| Maize | 18.6 | 20.4 | 15.3 | 35.9 | 13.9 | 18.2 | 13.8 | 24.2 | 16.2 | 17.2 | 7.8 | 10.8 | 26.6 | 12.8 | 12.4 | 4.8 | 0.8 | 3.9 |
| Oilseed rape | 2.1 | 0.4 | 0.0 | 0.8 | 0.0 | 0.1 | 4.2 | 0.0 | 0.0 | 1.2 | 0.0 | 0.9 | 0.0 | 0.0 | 0.5 | 4.5 | 0.0 | 0.0 |
| Onion | 0.7 | 3.0 | 0.0 | 0.0 | 0.3 | 2.7 | 0.0 | 0.4 | 0.0 | 2.6 | 0.0 | 1.3 | 2.1 | 0.0 | 0.0 | 3.0 | 4.4 | 10.7 |
| Potato | 0.6 | 0.9 | 0.0 | 0.7 | 0.0 | 0.0 | 1.5 | 0.8 | 0.7 | 7.7 | 2.2 | 1.9 | 1.0 | 1.4 | 0.0 | 10.2 | 6.8 | 5.2 |
| Pumpkin | 2.1 | 3.8 | 0.5 | 3.8 | 1.3 | 5.3 | 1.0 | 0.8 | 2.5 | 0.7 | 2.9 | 3.1 | 2.7 | 0.7 | 0.5 | 8.2 | 8.2 | 4.0 |
| Radish | 0.2 | 2.3 | 1.4 | 0.3 | 0.0 | 0.8 | 3.2 | 1.0 | 0.0 | 0.0 | 1.1 | 1.4 | 0.0 | 0.0 | 0.0 | 3.3 | 4.5 | 0.7 |
| Rhubarb | 0.9 | 1.2 | 0.0 | 0.0 | 1.9 | 0.1 | 0.0 | 0.7 | 1.1 | 0.0 | 0.9 | 0.0 | 0.0 | 0.0 | 0.0 | 0.2 | 0.0 | 1.0 |
| Salad | 0.1 | 3.4 | 0.0 | 0.6 | 0.7 | 1.7 | 0.0 | 0.0 | 0.0 | 0.0 | 1.4 | 3.0 | 0.6 | 0.0 | 0.0 | 0.0 | 0.2 | 2.0 |
| Strawberry | 0.0 | 0.4 | 0.0 | 0.0 | 0.4 | 2.0 | 0.0 | 0.0 | 4.0 | 0.0 | 0.4 | 1.5 | 0.0 | 0.0 | 0.0 | 0.0 | 1.1 | 0.0 |
| Sugar beet | 0.3 | 5.5 | 3.6 | 18.2 | 0.0 | 5.9 | 2.4 | 7.5 | 3.1 | 12.9 | 4.4 | 8.6 | 3.6 | 3.7 | 0.3 | 3.1 | 1.9 | 3.3 |
| Vine | 12.6 | 8.7 | 0.0 | 6.4 | 0.0 | 0.1 | 0.0 | 1.0 | 0.9 | 2.1 | 2.2 | 0.0 | 1.5 | 0.0 | 0.0 | 0.0 | 0.0 | 0.0 |
| Winter cereals | 11.3 | 7.5 | 13.2 | 12.9 | 1.9 | 10.8 | 15.9 | 16.8 | 3.5 | 40.7 | 13.3 | 7.1 | 12.3 | 7.3 | 16.7 | 13.4 | 1.9 | 1.1 |
| Other crops | 5.8 | 20.0 | 4.2 | 9.1 | 6.0 | 12.6 | 14.1 | 4.3 | 4.8 | 3.5 | 14.0 | 5.9 | 8.8 | 0.8 | 1.2 | 14.9 | 12.2 | 11.3 |
| **Cropland** | 60.9 | 90.7 | 38.1 | 89.8 | 28.0 | 72.6 | 65.1 | 62.9 | 39.3 | 90.8 | 51.3 | 48.5 | 61.8 | 33.8 | 31.6 | 67.6 | 50.3 | 50.1 |
| Ha | 22.5 | 0.6 | 23.1 | 3.2 | 17.8 | 6.6. | 11.2 | 3.3. | 7.3 | 1.5 | 23.1. | 7.8 | 7.8 | 0.5 | 8.4 | 6.0 | 0.4 | 2.8 |
| hl | 2.5 | 1.6 | 1.6 | 2.0 | 1.8 | 1.8 | 2.6 | 1.2 | 2.6 | 2.7 | 1.7 | 1.4 | 2.8 | 2.5 | 2.6 | 3.0 | 1.5 | 1.7 |
| wa | 3.2 | 0.0 | 1.2 | 0.0 | 24.2 | 9.7 | 9.3 | 15.4 | 20.8 | 0.0 | 2.8 | 37.9 | 7.5 | 6.7 | 17.7 | 3.7 | 4.1 | 0.0 |
| wl | 3.7 | 2.8 | 6.3 | 2.3 | 3.9 | 3.4 | 4.0 | 0.5 | 4.8 | 2.1 | 4.3 | 2.3 | 1.7 | 4.9 | 4.6 | 5.0 | 3.5 | 3.6 |
| **SNH** | 31.9 | 5.0 | 32.9 | 7.4 | 47.7 | 21.6 | 27.1 | 20.4 | 35.5 | 6.3 | 31.8 | 49.4 | 19.8 | 14.7 | 33.3 | 17.7 | 9.4 | 8.1 |
| urban | 4.9 | 2.2 | 25.1 | 0.7 | 20.9 | 2.7 | 3.4 | 15.1 | 23.0 | 0.0 | 14.9 | 0.3 | 12.0 | 28.0 | 25.4 | 6.5 | 33.7 | 37.0 |
| Water bodies | 0.3 | 0.0 | 0 | 0.0 | 0.0 | 0.0 | 0.1 | 0.0 | 0.2 | 0.0 | 0.0 | 0.3 | 4.6 | 21.6 | 5.9 | 0.2 | 0.5 | 0.4 |
| Other habitats | 2.0 | 2.1 | 4.9 | 2.1 | 3.3 | 3.1 | 4.3 | 1.6 | 1.9 | 2.9 | 1.5 | 1.6 | 1.8 | 1.9 | 3.8 | 8.0 | 6.2 | 4.4 |

**Table S2**

Average number of insecticide treatments of the 16 dominant crops in our study area (proportion of total area in the landscapes, LS = number of landscapes, where the crop was present) according to the cited literature.

| **crop** | **% of total area (range)** | **LS** | **N insecticide treatments** | **literature** |
| --- | --- | --- | --- | --- |
| apple | 0 – 10% | 9 | 7.5 | Roßberg 2009 |
| asparagus | 0 – 5% | 10 | 1.1 | Roßberg & Hommes 2014 |
| cabbage | 0 – 4% | 10 | 4.9 | Roßberg & Hommes 2014 |
| carrot | 0 – 7% | 11 | 1.7 | Roßberg & Hommes 2014 |
| maize | 1 – 36% | 18 | 0.03 | Roßberg 2016 |
| oilseed rape | 0 – 4% | 9 | 2.7 | Roßberg 2016 |
| onion | 0 – 11% | 11 | 0.7 | Roßberg & Hommes 2014 |
| potato | 0 – 10% | 14 | 0.8 | Roßberg 2016 |
| pumpkin | 1 – 8% | 18 | 0.5 | Own data mean of 18 conv. fields |
| raphanus | 0 – 4% | 11 | 2.0 | DLR, pers. comm. |
| rhubarb | 0 – 2% | 10 | 0.0 | DLR, pers. comm. |
| salad | 0 – 3% | 10 | 2.7 | Roßberg & Hommes 2014 |
| strawberry | 0 – 4% | 7 | 2.4 | Roßberg 2009 |
| sugar beet | 0 – 18% | 17 | 0.14 | Roßberg *et al.* 2010 |
| vine | 0 – 14% | 9 | 0.4 | Roßberg 2010 |
| winter wheat | 1 – 41% | 18 | 0.7 | Roßberg 2016 |

**References**

DLR (Dienstleistungszentrum ländlicher Raum), Weinheimer, S., personal communication.

Roßberg, D. (2009). NEPTUN 2007 - Obstbau. Julius Kühn Institute, Federal Research Centre for Cultivated Plants, Braunschweig, Germany. pp. 175.

Roßberg, D. (2010). Neptun 2009 - Weinbau. Julius Kühn Institute, Federal Research Centre for Cultivated Plants, Braunschweig, Germany. pp. 151.

Roßberg, D. (2016). Erhebungen zur Anwendung von Pflanzenschutzmitteln im Ackerbau. Survey on application of chemical pesticides in agriculture. *Journal für Kulturpflanzen*, 68, 25–37.

Roßberg, D. & Hommes, M. (2014). NEPTUN-Gemüsebau 2013*.* Julius Kühn Institute, Federal Research Centre for Cultivated Plants, Braunschweig, Germany. pp. 175.

Roßberg, D., Vasel, E.-H. & Ladewig, E. (2010). NEPTUN 2009 - Zuckerrübe. Julius Kühn Institute, Federal Research Centre for Cultivated Plants, Braunschweig, Germany. pp. 152.

**Table S3** Pearson rank correlation coefficients for all pairs of explanatory variables (lower panel) and asymptotic p-values (upper panel). Pearson correlations with r ≥ 0.40 (p ≤ 0.1) are marked in bold. Variables with r > 0.6 are not included in the same model. For variables with r < 0.4 covariances were fixed in the structure equation model.

|  | Adjacent SNH | Organic | % Agricultural land | % SNH | Insecticide intensity |
| --- | --- | --- | --- | --- | --- |
| Adjacent SNH |  | 1.0 | 0.08 | 0.61 | 0.04 |
| Organic | 0 |  | 1.0 | 0.52 | 0.93 |
| % Cropland | **-0.42** | 0 |  | 0.004 | 0.10 |
| % SNH | 0.13 | -0.16 | **-0.65** |  | 0.11 |
| Insecticide intensity | **-0.48** | -0.02 | 0.40 | -0.39 |  |

**Table S4** Direct effects of adjacent habitat (factor: crop or SNH), field management (factor: organic or conventional), proportion of seminatural habitats in 1 km radius (% SNH, continuous), and insecticide intensity in the landscape (continuous) on visits of honey and bumble bees and direct and indirect effects of them on pollen delivery. Indirect effects on pollen delivery are split in effects mediated by bumble bee visits or by honey bee visits. Results from the structure equation model (number of observations = 18, minimum generalised least-squares chi-square statistic = 12.5, df = 11) are displayed. For all predictors estimates, standard errors, z-values and p-values are given. R^2^ is given per response.

| **response** | **mediated by** | **predictor** | **estimate** | **Std.Err** | **z-value** | **p** | **R^2^** |
| --- | --- | --- | --- | --- | --- | --- | --- |
| Honey bee visits |  | ~ |  |  |  |  | 0.19 |
|  |  | Adjacent SNH | -1.4 | 20.2 | -0.07 | 0.95 |  |
|  |  | Organic | -18 | 11 | -1.7 | 0.096 |  |
|  |  | % SNH | 0.4 | 0.6 | 0.7 | 0.51 |  |
|  |  | Insecticide intensity | -1.7 | 5.2 | -0.3 | 0.75 |  |
| Bumble bee visits |  | ~ |  |  |  |  | 0.51 |
|  |  | Adjacent SNH | 12 | 10 | 1.2 | 0.24 |  |
|  |  | Organic | 7.3 | 5.6 | 1.3 | 0.20 |  |
|  |  | % SNH | 0.6 | 0.3 | 1.9 | 0.050 |  |
|  |  | Insecticide intensity | 1.8 | 2.5 | 0.7 | 0.48 |  |
| Pollen delivery |  | ~ |  |  |  |  | 0.82 |
|  |  | Honey bee visits | 6 | 21 | 0.3 | 0.77 |  |
|  |  | Bumble bee visits | 255 | 51 | 5.0 | < 0.001 |  |
|  | Honey bee visits | Adjacent SNH | -8 | 125 | -0.07 | 0.95 |  |
|  |  | Organic | -112 | 394 | -0.3 | 0.78 |  |
|  |  | % SNH | 2 | 9 | 0.3 | 0.80 |  |
|  |  | Insecticide intensity | -10 | 46 | -0.2 | 0.82 |  |
|  | Bumble bee visits | Adjacent SNH | 3052 | 2567 | 1.2 | 0.23 |  |
|  |  | Organic | 1859 | 1382 | 1.3 | 0.18 |  |
|  |  | % SNH | 152 | 74 | 2.1 | 0.040 |  |
|  |  | Insecticide intensity | 460 | 651 | 0.7 | 0.48 |  |

**Table S5** Number and mode of field operations (number of herb management operations (mechanical and herbicide applications), number of applications of insecticides and fungicides, and the amount of nitrogen in kg/ha) are based on farmer´s questionaires. The proportion of weed cover was measured in four 5m^2^-plots per field at four samplings (28.-30.5., 25.-27.6., 15.-17.7. and 6.-9.8.2014). The diversity and abundance of flowering weeds was measured in twenty 1m^2^- plots per field at four samplings (28.-30.5. and parallel to the three pollination samplings on 2-6, 15-17, 23-25 of July 2014). Superscript letters (A, B) mark significant different groups. The test statistic (F- or χ^2^-value with degrees of freedom and p-value) are given.

| **variable** | **organic** | | **conventional** | **value** | **p** |
| --- | --- | --- | --- | --- | --- |
|  | EU-Bio | Bio-association |  |  |  |
| N fields | 3 | 6 | 9 |  |  |
| N herb management | 2.3^A^ | 2.3^A^ | 3.3^B^ | F_1,16_ = 7.2 | 0.016 |
| Mode herb management | mechanical | | Herbicides (1.4)  + mechanical (1.9) | |  |
| % weed cover | 2.3^A^ | 4.8^A^ | 8.5^B^ | χ^2^_1,16_ = 3.9 | 0.049 |
| N flowering weeds (log) | 1.8 | 1.7 | 2.5 | F^2^_1,16_ = 2.5 | 0.13 |
| S flowering weeds | 4.3 | 4.8 | 6.8 | χ^2^_1,16_ = 1.5 | 0.22 |
| N insecticides | 3^A^ | 0^B^ | 0.3^B^ | χ^2^_2,14_ = 19 | < 0.001 |
| N fungicides | 2.3^A^ | 0^B^ | 2^A^ | χ^2^_2,14_ = 32 | < 0.001 |
| Nitrogen [kg/ha] | 142^A^ | 76^B^ | 103^AB^ | F_2,14_ = 3.6 | 0.054 |
| Fertilizer mode | organic | | synthetic |  |  |
